# Monitoring in the Intensive Care Unit: Its Past, Present, and Future

**DOI:** 10.1155/2012/452769

**Published:** 2012-09-17

**Authors:** Maxime Cannesson, Alain Broccard, Benoit Vallet, Karim Bendjelid

**Affiliations:** ^1^Department of Anesthesiology and Perioperative Care, University of California Irvine, Irvine, CA 92697, USA; ^2^Division of Pulmonary and Critical Care Medicine, Department of Medicine, University of Minnesota, Minneapolis, MN 55435-2199, USA; ^3^Department of Anesthesiology and Critical Care Medicine, University Hospital of Lille Nord de France, 59037 Lille, France; ^4^Intensive Care Division, Geneva Medical School, Geneva University Hospitals, 1211 Geneva 14, Switzerland

Monitoring in the critical care setting has dramatically improved during the past 50 years and has contributed significantly toimprove patients' safety and outcome [1–3]. New technologies have allowed the transfer ofadvances in biology, physiology, and bioengineering to the bedside to support data drivendecision making and continuous monitoring of the vulnerable critically ill patients.The most striking advances include the continuous and noninvasive measurement of oxygen saturation by pulseoximeters and of end tidal CO_2_ and the real-time displays of flow, volume, pressure time curves, and derived measures by modern ventilators as well as the development invasive and more recently noninvasive devices that provide beat-to-beat arterial pressure, stroke volume, and cardiac output monitoring.

Despite these advances and the apparent impact made on patients' outcome, there are still a lot of progress to be made to bring monitoring to the level of safety and reliability achieved by industries such as aviation [3, 4].

The future of monitoring in the critical care setting probably relies less on global appraisal of descriptive variables and more on functional monitoring of organs. Ultimately monitoring complex organ function is more informative and will likely be more important than global and/or regional physiological parameterssuch as organs perfusion and oxygenation. Metabolic monitoring, reflecting the biologic functions of the organs, starts to emerge [5]. Noninvasive monitors and trend analysis will obviously continue to grow. In addition, more advanced monitoring of pain, sleep, wakefulness, and delirium are very much needed.Atthe end of the day, decision support systems and automated system will become instrumental and central in daily monitoring when such system can provide the high level of accuracy needed to allow health care providers to rely on them [6, 7]. In addition, decision support systems willonly make sense if they improveclinicians' decision making, not if they just synthesized clinical algorithms. We expect that decision support software that integrates monitoring signals to raise the safety, reliability, and efficiency bar and not to fully replace human being. Finally, there is still a lot to be learned regarding identifying which variables should be monitored to impact outcome and what constitute an appropriate as oppose to pathological harmful one to critical illnesses. Without such understanding, enhanced monitoring has the potential to lead to costly and counterproductive interventions.

Finally, one has to ask whether new monitoring technologies must be evaluated and clearly demonstrate a positive impact on outcome before being used. There is no easy and universal answer to this question, we believe. Most hospital administrators may require outcome data before purchasing any new and potentially expensive technologies. This approach could, however, delay the implementation of useful technologies. It is indeed possible and likely that initial studies, even when well conducted, could only show no impact on outcome [8]. As an example, the pulse oximeter has been shown to have no impact on patients outcome [9, 10] despite the fact that this is considered standard of care. While some in the medical community are still wondering whether pulse oximeters do improve outcome since the data is lacking, in other industries such as aviation evidence-based data before implementing new technologies (monitors, autopilot, simulation) is not required and this industry has now reached an unmatched level of safety. On the other end, a more thoughtful assessment of clinical indication and physician education of physicians regarding Swan-Ganz catheter and hemodynamic management would have prevented many unhelpful right heart catheter placement over decades and possible harm. Clearly, there is not a single simple answer for every technology and/or problem at hand.

In conclusion, monitoring in our specialty has come a long way. We are, however, still facing difficult challenges and the future holds great promisesfor our patient [3], particularly if, as an scientific community, we can learn from our past mistakes.This special issue on monitoring of critical patients illustrates some of the current and future challenges we are facing.

*Maxime  Cannesson* *Alain  Broccard* *Benoit  Vallet* *Karim  Bendjelid*
